# Characterization of *Brassica rapa* RAP2.4-Related Proteins in Stress Response and as CUL3-Dependent E3 Ligase Substrates

**DOI:** 10.3390/cells8040336

**Published:** 2019-04-10

**Authors:** Sutton Mooney, Raed Al-Saharin, Christina M. Choi, Kyle Tucker, Chase Beathard, Hanjo A. Hellmann

**Affiliations:** School of Biological Sciences, Washington State University, Pullman, WA 99164, USA; suttonmooney@wsu.edu (S.M.); Raed.alsaharin@wsu.edu (R.A.-S.); christina.choi@wsu.edu (C.M.C.); kyletucker67@gmail.com (K.T.); chase.beathard@wsu.edu (C.B.)

**Keywords:** RAP2.4, stress, proteasome, transcription, PEST

## Abstract

The turnip *Brassica rapa* has important economic value and represents a good model system to study gene function in crop plants. ERF/AP2 transcription factors are a major group of proteins that are often involved in regulating stress-responses and developmental programs. Some ERF/AP2 proteins are targets of CULLIN3-based E3 ligases that use BTB/POZ-MATH proteins as substrate receptors. These receptors bind the transcription factor and facilitate their ubiquitylation and subsequent degradation via the 26S proteasome. Here, we show tissue and stress-dependent expression patterns for three *Brassica rapa* ERF/AP2 proteins that are closely related to *Arabidopsis thaliana* AtRAP2.4. Cloning of the *Brassica* genes showed that the corresponding proteins can assemble with a BPM protein and CULLIN3, and that they are instable in a 26S proteasome dependent manner. This work demonstrates the conserved nature of the ERF/AP2-CULLIN3-based E3 ligase interplay, and represents a first step to analyze their function in a commercially relevant crop plant.

## 1. Introduction

ETHYLENE RESPONSE FACTOR/APETALA2 (ERF/AP2) transcription factors are key regulators of developmental and physiological responses in plants, and they are also often connected with abiotic stress control [1,2,3]. With increasingly rapid environmental changes and continued population growth, it has become even more important to understand the regulatory aspects that control their activity in planta. 

One ERF/AP2 transcription factor in Arabidopsis that has recently been brought into context with phytohormone signaling, wound-response, and abiotic stress control is related to APETALA2.4/WOUND INDUCED DEDIFFERENTIATION1 (RAP2.4/WIND1/At1g78080, further referred to as At RAP2.4) [4,5,6,7]. In Arabidopsis, the gene is most strongly expressed in root and dry seeds but can be strongly induced throughout the plant by abiotic stress treatments, such as drought and salt, or by the phytohormone abscisic acid [6,7,8]. Consequently, overexpression of the transcription factor has been connected to increased drought tolerance and up-regulated expression of water-stress related genes in Arabidopsis and rice (*Oryza sativa*) [7,8,9]. However, overexpression of a RAP2.4-like protein from papaya (*Carica papaya* cv. Maradol) also confers cold and heat tolerance in tobacco (*Nicotiana tabacum*) [9]. *AtRAP2.4* is also strongly up-regulated after wounding, and high levels of transcription promote cell dedifferentiation and cell proliferation [5,10]. This function of AtRAP2.4 appears to be conserved among different plant species, as its overexpression in tomato, tobacco, and *Brassica napus* also results in enhanced cell dedifferentiation and callus formation, as first observed in Arabidopsis [4,11]. Recent work from Iwase and co-workers also identified a direct target gene of AtRAP2.4 that is called ENHANCER OF SHOOT REGENERATION1 (*ESR1*), and is involved in normal shoot regeneration [12]. Metabolic analysis in *Brassica napus* hypocotyl explants overexpressing AtRAP2.4 leads to wide metabolic changes [13]. Interestingly, some of the metabolites that were consistently increased, such as γ-amino butyric acid and L-proline, have been brought into context with biotic and abiotic stress responses [13,14,15,16,17]. These findings may also explain why increased levels of RAP2.4 in different plant species, such as tobacco, *B. napus* or Arabidopsis, provide a better abiotic stress tolerance. 

We have previously demonstrated that members of the ERF/AP2 family in Arabidopsis are prone to degradation mediated by a CUL3-dependent RING E3 ligase (CRL3) that uses BTB/POZ-MATH (BPM) proteins as substrate receptors (CRL3^BPM^) [18,19,20,21,22]. E3 ligases are central regulatory bottlenecks within the ubiquitin proteasome pathway, which functions as a major degradation and signal transduction pathway in eukaryotic organisms [23]. BPM proteins appear to be conserved among the plant kingdom, but their absolute numbers can vary significantly across different plant species. While Arabidopsis expresses six BPM proteins, there are 11 in *B. rapa*, 31 in maize (*Zea mays*), and 76 members in rice [24,25,26]. A recent evaluation of the phylogenetic development of BPM proteins further indicated that eudicot and monocot plant species share a common core clade of BPM proteins, but that specifically, the grasses have independently expanded and multiplied these proteins, likely an adaptation to the specific needs of the *Poales* order [25]. BPM proteins have meanwhile been established as important regulatory proteins that control a broad range of transcriptional processes in Arabidopsis by controlling the stability of members from at least three different transcription factor families, ERF/AP2, MYB, and AtHB [18,19,27,28,29]. As a consequence, they are involved in abscisic acid signal transduction, heat and drought tolerance, stomatal movement, root and shoot development, seed fatty acid biosynthesis, and flower development [18,27,28,29]. 

Here we show that public databases predict at least three genes in *B. rapa* that are closely related to Arabidopsis *AtRAP2.4*. Cloning of these three genes revealed that one is identical, one is mildly different, and one has significant sequence divergence from the publicly available *B. rapa* sequence database. Expression analysis showed a distinct tissue and stress specific expression pattern for each of the three genes. All three BrRAP2.4-like proteins are able to assemble with an Arabidopsis BPM protein, and are instable in a proteasome-dependent manner. They also have a PEST motif in their C-terminal region, which they share with their ortholog from Arabidopsis, and which is often connected with instability in proteins. We demonstrate that the PEST motif is indeed critical for stability, and also for assembly with BPMs, but not for their subcellular localization. Expression of one *BrRAP2.4* member in Arabidopsis results in improved salt tolerance at the germination and seedling stage, supporting the notion that RAP2.4 proteins facilitate improved abiotic stress tolerance. 

## 2. Materials and Methods

For this work the Brassica A genome species *Brassica rapa ssp. oleifera* (variety R-o-18), *Arabidopsis thaliana* (variety Col-0), and *Nicotiana benthamiana* were used. For sterile culture, Arabidopsis thaliana salt (ATS) medium without supplemental sucrose was used [30], with plants growing under long-day conditions (16h:8h light:dark). Soil grown plants were cultivated in a growth room under standard conditions (20 °C, 60% humidity, 16 h:8 h light:dark). All Brassica stress treatments were performed with 7 day-old seedlings grown in sterile culture. For drought stress, culture plates were opened up for 30 min to expose seedlings to air, covered afterwards and allowed to recover for a 30 min period, before samples were taken. For wound response, sharp forceps were used to poke four holes in each leaf. Samples were harvested 1 h later. A 3 h heat stress was applied by transferring seedlings from culture plates to 37 °C preheated, liquid ATS medium. Flasks containing stressed seedlings were afterwards moved to 20 °C for a recovery period of 1 h, after which samples were taken. For cold stress, seedlings were transferred from plates to a 4 °C pre-chilled liquid ATS medium and incubated for 5 h, after which samples were directly taken. Salt and osmotic stress treatments were applied by transferring seedlings from culture plates to liquid ATS medium supplemented with or without NaCl (100 or 200 mM) or sorbitol (300 mM), respectively. 

For NaCl-dependent germination assays, transgenic and wild type Arabidopsis seeds were plated on ATS medium supplemented with different salt concentrations. Germination was defined when radicles first emerged from the seed coat. Salt-dependent root length assays were performed using Arabidopsis seedlings grown vertically on ATS medium. Three days post-germination, seedlings were transferred individually to fresh plates supplemented with or without different NaCl concentrations. Primary root length was measured daily for up to two weeks. 

Genomic DNA was isolated from *B. rapa* using standard procedures. PCR was done using Phusion High-Fidelity DNA Polymerase (Thermofisher). Specific primers for each *BrRAP2.4* gene were designed based on publicly available data to bind to the respective 5’ and 3’ ends (Table S1). PCR reactions were performed under standard conditions (25 cycles, 30 seconds at 55 °C annealing, 1 min elongation at 72 °C, 15 s denaturation at 94 °C). PCR products were sub-cloned into *pCR8* (LifeScience) and sequenced. For in vitro experiments, the three *BrRAP2.4* genes were shuffled via LR-GATEWAY-reactions to the GST-fusion vector *pDEST15* (LifeScience). Constructs were transformed into the *E. coli* strain Rosetta and expression was induced with IPTG (UBPBio) for two hours once the OD_600_ was between 0.7 and 0.8. Expressed proteins were affinity purified from *E. coli* via glutathione agarose beads (Sigma-Aldrich). *His:BPM3* was cloned into *pET21b* using the *Nde*I/*Xho*I restriction sites (Novagen), and purified from *E. coli* via Ni-NTA agarose beads (Sigma-Aldrich). For in planta expression studies, the different *BrRAP2.4* genes were cloned into the GATEWAY-compatible vector *pMDC43* [31] that permits expression of an N-terminally tagged GFP-fusion protein. Truncated *AtRAP2.4* and *BrRAP2.4-1* versions lacking their PEST motifs were generated through PCR, subcloned in pDONR^ZEOCIN^, sequenced, and shuffled into *pDEST15* and *pMDC43* for in vitro and in planta experiments, respectively. 

For transient expression analysis in *N. benthamiana* a basic protocol was followed according to Sparkes et al. (2006) [32]. Stable transformations into Arabidopsis were done using the floral dip method as described in Clough and Bent (1998) [33].

Total *B. rapa* and Arabidopsis RNA extraction was done using an Isolate RNA kit from Bioline, following the manufacturer’s protocol. A high-capacity cDNA reverse transcription kit from Applied Biosystems was used to perform reverse transcription reactions. qRT-PCR reactions were done under standard conditions on a 7500 Fast Real-Time PCR system (Applied Biosystems) as described earlier [18]. Calculation of gene expression was done with *ACTIN2* as the internal control gene. Unless otherwise indicated, at least three biological replicates were performed for each individual experiment. Primers used for qRT-PCR are listed in Table S1.

Cell-free degradation assays were done according to Wang et al. (2009) and Lu et al. (2010) [34,35]. Two week-old sterile grown Arabidopsis seedlings were used for total protein plant extract. *E. coli* expressed and purified GST-recombinant proteins were eluted from glutathione agarose beads and checked by silver staining SDS-PAGE. Incubation of recombinant protein in plant extract was performed in Eppendorf tubes on a room temperature rocker, from which 20 uL samples were withdrawn at the indicated time points. Incubation with the proteasomal inhibitor MG132 was done simultaneously in separate tubes. Upon collection, samples were added to tubes prepared with protein loading dye, boiled, and frozen. Standard Western blot analysis was performed with monoclonal GST antibodies purchased from LifeTein, NJ, and horseradish-coupled secondary donkey anti-mouse from Santa Cruz, CA.

Pulldown assays were done with the *E. coli* expressed and purified recombinant GST:RAP2.4 and His:BPM3 proteins. Total protein plant extracts and pulldown assays were done as described earlier [18,19]. In brief, purified recombinant proteins were analyzed on SDS-PAGE gel by silver-staining and Western blot to ensure quality and compare input concentrations prior to experimental assays. For pulldown assays, GST-proteins on beads were incubated with eluted His-protein, or GST-proteins were eluted and incubated with His-protein on beads, in 300 μL pulldown buffer (100 mM Tris/HCL pH 7.5, 150 mM NaCl, 0.1% Tween-20) rocking at 4 °C for one hour. This was followed by three washes (10 min, rocking at 4 °C) where proteins were in between briefly spun down (1000 rpm, 30 sec), then the supernatant was taken off and replaced with 1ml pulldown buffer. After washes, samples were directly taken up in Laemmli loading buffer, heated for 5 min at 95 °C, and used for SDS-PAGE, Western blot, and immunodetection. For in planta detection of CUL3, plant extracts were generated by grinding two-week old Arabidopsis seedlings grown in sterile culture in extraction buffer, quantified by Bradford assay on a spectrophotometer (Amersham Ultrspec 2100pro), and adjusted for a final concentration of 3mg/mL. Western blot analysis was done using standard procedures. Custom made polyclonal antibodies against CUL3 [18] were generated by GenScript, NJ. Monoclonal GST and His-antibodies were purchased from LifeTein, NJ, USA, while horseradish-coupled secondary donkey anti-mouse and anti-rabbit antibodies came from Santa Cruz, CA, USA.

For qRT-PCR analysis, samples were normalized against *ACTIN2* and relative gene expression was calculated using the ΔΔCt method described in [36], and further described in each respective legend. Student’s t-tests were performed in Microsoft Excel to analyze significance of qRT-PCR data. Protein quantification was done using ImageJ and significance was determined using one-way ANOVA tests from online tools (https://www.socscistatistics.com/tests/anova/default2.aspx).

Confocal microscopy was done using a Leica SP-8 Confocal Laser Scanning Microscope (Leica Microsystems Inc., Buffalo Grove, IL, USA), and standard methods as described earlier [37]. 

## 3. Results

### 3.1. Brassica Rapa Encodes for Three RAP2.4-Like Genes

BLAST searches with the Arabidopsis AtRAP2.4 protein against the publicly available *B. rapa* (Chiifu-401 variety) database, (BRAD; http://brassicadb.org), yielded several proteins with a high similarity to Arabidopsis. Most were significantly shorter than AtRAP2.4, with less than 260 amino acids and identity scores lower than 60% to AtRAP2.4. The shorter proteins resembled AtRAP2.13/AtRAP2.4b/AtWIND2/At1g22190, the closest relative to AtRAP2.4 in Arabidopsis [19,38]. 

**Figure 1:**
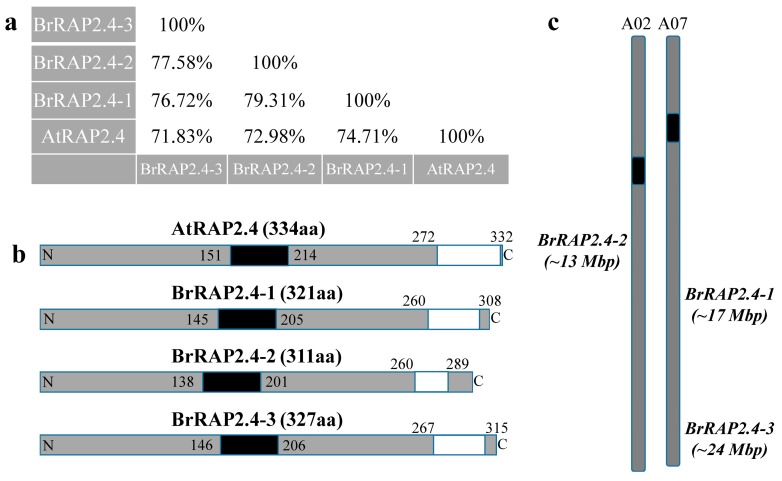
Comparison of Arabidopsis and *B. rapa* RAP2.4-like proteins. (**a**) Degrees of identity of the amino acid sequences from AtRAP2.4 and three orthologs from *B. rapa.* Analyses were performed online using the ClustalW program for sequence alignment, and the SIAS tool (http://imed.med.ucm.es/Tools/sias.html) for identity scores. (**b**) Schematic drawing of RAP2.4 proteins from Arabidopsis and *B. rapa* showing their length and the positions of the AP2 DNA binding domain (black box), and the predicted PEST motifs (white box). (**c**) *B. rapa* chromosome A02 and A07 with the predicted locations of *BrRAP2.4-1* to *-3*, respectively.

We focused on the three annotated *B. rapa* proteins that were of comparable length to AtRAP2.4, and which showed identities greater than 70% as criteria to classify these as AtRAP2.4-like candidates (Figure 1a). Based on the degree of identity to AtRAP2.4, we named the corresponding proteins BrRAP2.4-1 (Bra003659), BrRAP2.4-2 (Bra008343), and BrRAP2.4-3 (Bra015634). Using the ELM server program (http://elm.eu.org/), a single AP2 DNA-binding domain was predicted for each of the BrRAP2.4 proteins roughly located close to their centers (Figure 1b). According to the epestfind program (http://emboss.bioinformatics.nl/cgi-bin/emboss/epestfind), AtRAP2.4 and all three BrRAP2.4 proteins also contained a PEST motif at their C-terminal ends. PEST motifs are often connected with protein instability [39,40,41,42] (Figure 1b). 

The *BrRAP2.4-1* (963 bp) and *-3* (981 bp) genes are both located on chromosome A07 at nucleotide positions 17,580,986 to 17,581,942 and 24,922,473 to 24923459, respectively, while *BrRAP2.4-2* (933 bp) is located on chromosome A02 between the nucleotides 13,877,035 to 13,877,970 (Figure 1c). As with AtRAP2.4, none of the three genes contain predicted introns.

### 3.2. Tissue Specific Expression of BrRAP2.4-Like Genes

**Figure 2:**
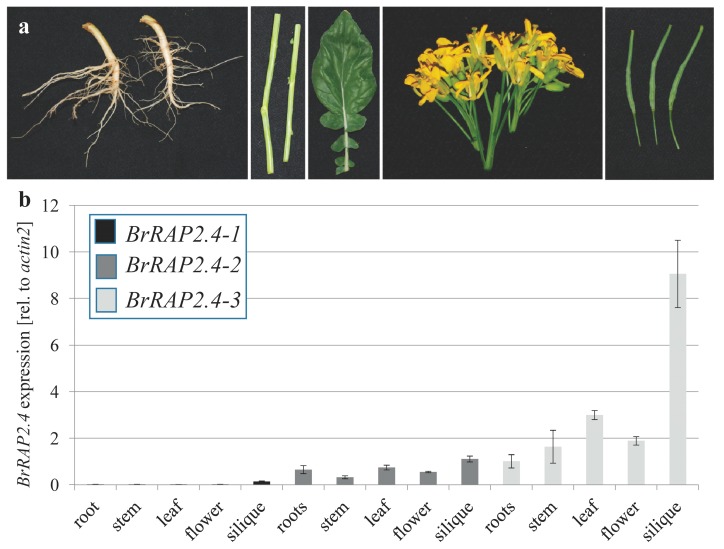
Tissue specific expression patterns of *BrRAP2.4-1* to *-3* genes. (**a**) Representative pictures of tissues used for qRT-PCR (from left to right: root, stem, source leaf, flowers, and siliques). (**b**) qRT-PCR analysis for *BrRAP2.4-1* to *-3*. Shown is a technical triplicate with standard error. For each sample, 90 ng of total RNA was used. Samples were normalized and compared against *ACTIN2.* A second biological replicate is shown in Supplementary Figure S1.

To verify that the annotated genes are indeed expressed and do not represent pseudogenes, we performed qRT-PCR analysis on different tissues (root, stem, source leaf, flower, and silique) from soil grown *B. rapa* (Figure 2; Supplementary Figure S1). As shown in Figure 2, all three *BrRAP2.4* genes are expressed, and show distinct patterns in the different tissues tested compared to one another. *BrRAP2.4-1* was expressed in general at very low levels and in all tissue. Its expression was either not detectable or close to the detection limit, with the highest levels in siliques. In contrast, *BrRAP2.4-3* was present to higher amounts in all tested tissues, but like *BrRAP2.4-1* most prominently in siliques. *BrRAP2.4-2* showed a nearly even expression pattern throughout the tested tissues, with intermediate levels compared to *BrRAP2.4-1* and *-3* (Figure 2; Supplementary Figure S1). Overall these data confirmed that all three *BrRAP2.4* genes are expressed in *B. rapa*.

### 3.3. BrRAP2.4-Like Expression in Response to Abiotic Stress

**Figure 3:**
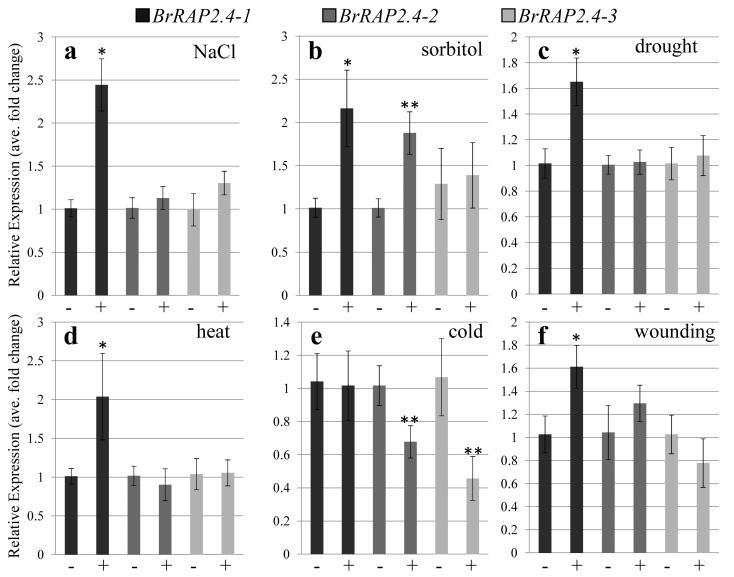
Abiotic stress-dependent expression of *BrRAP2.4-1* to *-3*. (**a**) Salt stress (NaCl), (**b**) Osmotic stress (sorbitol). (**c**) Drought stress. (**d**) Heat stress. (**e**) Cold stress. (**f**) Wound response. For each sample, 90 ng of total RNA was used. Calculations were normalized against *ACTIN2* as an internal control and compared to the *BrRAP2.4* untreated samples. Graphs represent averages of at least *n* = 3 biological replicates. Error bars represent standard deviation. Significant changes (student’s *t*-test) are indicated by asterisks (* *p* < 0.05; ** *p* < 0.01). −, no stress treatment; +, stress treatment.

*AtRAP2.4* is regulated by a broad range of abiotic stress stimuli [5,6,7]. To investigate whether this also holds true for its three orthologs in *B. rapa*, we performed a variety of stress tests including salt, heat, cold, drought, osmotic, and wound stress response. Interestingly, while hardly any expression was detectable for *BrRAP2.4-1* in the different tissues, the gene was up-regulated under all the different stress treatments except for cold (Figure 3), indicating that *BrRAP2.4-1* mainly plays a role in stress alleviation. Notably, both *BrRAP2.4-2* and *-3* were repressed under cold stress by around 40% and 50%, respectively (Figure 3). *BrRAP2.4-2* was mildly up-regulated by sorbitol, while *BrRAP2.4-3* had a tendency to be repressed by wounding (Figure 3). However, overall *BrRAP2.4-2* and *-3* were not at all or only weakly responsive to most of the stress treatments tested. 

### 3.4. Cloning of BrRAP2.4-1 and -3 Showed Differences to Annotated Sequences

In an attempt to get a better understanding of the BrRAP2.4 proteins, we decided to clone the corresponding genes. Since they do not contain introns, we amplified the open reading frames directly from genomic DNA. Products of the right size were obtained, and after sequencing it was determined that *BrRAP2.4-2* was identical with the publicly available sequence (Supplementary Figure S5a). However, *BrRAP2.4-1* (Supplementary Figure S2) and *BrRAP2.4-3* (Supplementary Figure S5b) showed some deviation from the public database that also leads to differences in the protein sequence. While BrRAP2.4-3 was only lacking a glycine (G) at amino acid position 214 in comparison to the annotated sequence, BrRAP2.4-1 deviated significantly from the publicly annotated *B. rapa* data (Supplementary Figure S3a,b). However, blast attempts using our sequence information against the BRAD database for *B. rapa* proteins, still resulted in Bra003659 as the closest homolog. 

To better understand this potentially significant sequence discrepancy observed for Bra003659, a general blast comparison against the NCBI database was performed. Interestingly, our clone from the *B. rapa* variety R-o-18 showed a 100% match to the *B. napus* protein BnaA07g20720D (Supplementary Figure S4). Because the qRT-PCR primers designed to test expression of the gene are fully identical with the annotated *Bra003659* sequence, and since *B. napus* is an allopolyploidic hybrid between *B. rapa* and *B. oleracea*, we considered it likely that the publicly available *B. napus* sequence mainly reflects the *B. rapa* ancestry. We decided to keep the name *BrRAP2.4-1* for the cloned gene from *B. rapa*. 

### 3.5. All BrRAP2.4 Genes Are Instable in a Proteasome-Dependent Manner

**Figure 4:**
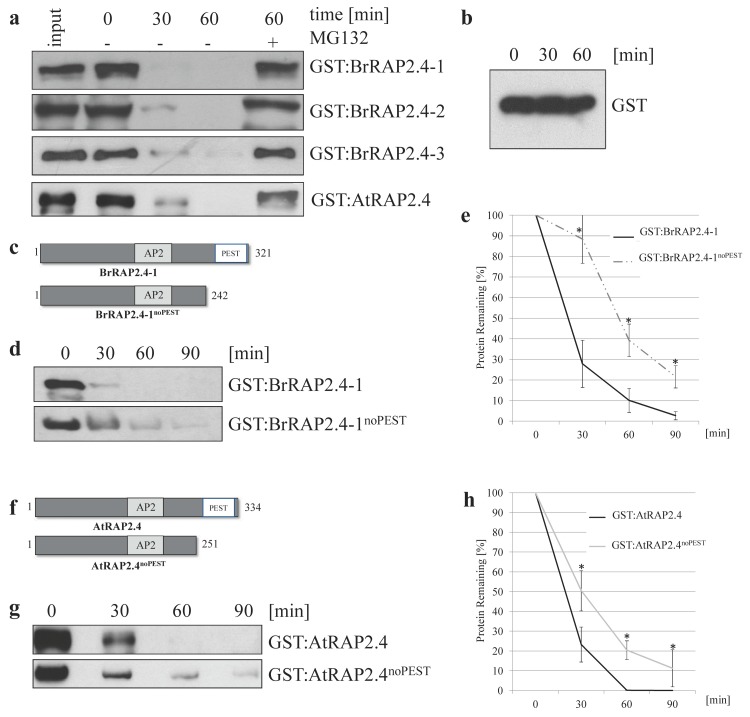
Characterization of protein stability for BrRAP2.4-1, -2, and -3. (**a**) Cell-free degradation assays demonstrate that all three BrRAP2.4 proteins are instable. Their degradation is a 26S proteasome dependent process since presence of MG132 in the assay stabilized the proteins. −, no MG132 treatment; +, MG132 treatment. (**b**) GST as a control is stable in the cell-free degradation assay. (**c**) schematic drawing of BrRAP2.4-1 and the noPEST deletion construct. (**d**) Western blot showing that BrRAP2.4^noPEST^ mutant protein remains longer detectable in a cell-free degradation assay then a full-length wild type version. (**e**) quantification of protein loss over time shows significantly higher levels of the noPEST version present in the assay compared to full-length BrRAP2.4-1; *n* = 6 biological replicates. (**f**) Schematic drawing of AtRAP2.4 and its noPEST version. (**g**) In a cell free degradation assay, AtRAP2.4^noPEST^ is more stable compared to AtRAP2.4. (**h**) Quantification of stability shows AtRAP2.4^noPEST^ is significantly more stable than its full-length version; *n* = 4 biological replicates. Asterisk indicate significance *p* < 0.05 based on an ANOVA test.

To test whether the BrRAP2.4 proteins are instable in a proteasome-dependent manner, we used a cell-free degradation assay [34] with total protein extracted from two week old, sterile grown Arabidopsis seedlings. AtRAP2.4 was included as a positive control, as previous results had already indicated its proteasomal degradation in planta [19]. Incubation of either GST:RAP2.4 protein showed their rapid degradation within 30 min, while treatment with the proteasomal inhibitor MG132 (20 μM) stabilized the proteins (Figure 4a). In comparison, GST alone was stable (Figure 4b), showing that the tested RAP2.4 proteins are unstable in a proteasome-dependent manner.

AtRAP2.4 and the three BrRAP2.4 proteins contain PEST motifs at their C-termini (Figure 1b). Because a PEST motif is often connected with protein instability [41,43,44], we were interested in whether deletion of that motif would result in stable proteins, and tested this for proof of principle for AtRAP2.4 and BrRAP2.4-1 (further referred to as AtRAP2.4^noPEST^ and BrRAP2.4-1^noPEST^; Figure 4c,f). 

As shown in Figure 4, the deletion of the PEST motif does not fully stabilize either protein, but results in a significant delay of their degradation (Figure 4d,e,g,h). These findings demonstrate that the PEST motif plays a critical role in controlling RAP2.4 stability in both Arabidopsis and *B. rapa.* Because the truncated proteins are still degraded, one can expect that additional degron motifs are present in AtRAP2.4 and BrRAP2.4-1. 

### 3.6. All BrRAP2.4 Proteins Assemble with Arabidopsis BPM3

**Figure 5:**
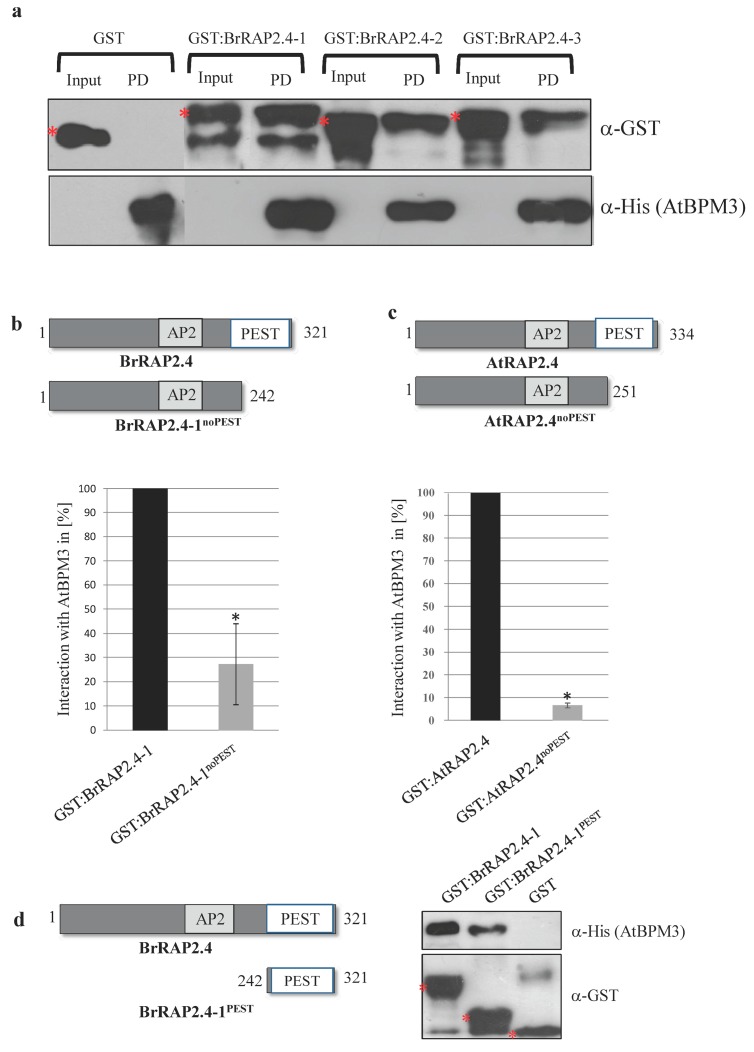
Characterization of protein assembly for BrRAP2.4-1, -2, and -3. (**a**) Pulldown assays with purified, recombinant His:AtBPM3 protein on beads resulted in co-precipitation of eluted GST:BrRAP2.4 proteins, while GST alone did not. Upper lane: Western-blot using an α-GST antibody, lower lane: stripped, and re-probed blot with an α-His antibody. (**b**) Upper part: schematic drawing of BrRAP2.4-1 and BrRAP2.4-1^noPEST^, and lower part: quantification of reduced interaction of GST:BrRAP2.4-1^noPEST^ with His:AtBPM3 compared to full-length BrRAP2.4-1. BrRAP2.4-1/AtBPM3 interaction was set to 100%, and BrRAP2.4-1^noPEST^/AtBPM3 put in relation to that. Data represent the average of *n* = 12 biological replicates. (**c**) Schematic drawing of AtRAP2.4 and AtRAP2.4^noPEST^ (upper part), and quantification of AtRAP2.4/AtBPM3 interaction as described in (**b**). Data represent average of *n* = 5 biological replicates. Asterisks in (**b**) and (**c**) indicate *p* < 0.05 significance based on an ANOVA test. (**d**) Pulldown analysis with GST:BrRAP2.4 proteins on beads resulted in co-precipitation of eluted His:AtBPM3 protein, shows that the BrRAP2.4 PEST motif alone is sufficient to bind AtBPMs. Red asterisks in (**a**) and (**d**) indicate full-length GST and GST-fusion proteins, respectively. PD, pulldown.

We further tested whether the BrRAP2.4 proteins are able to assemble with BPM proteins by performing in vitro pulldown assays using recombinant GST, GST:BrRAP2.4, and His:AtBPM3 proteins. As shown in Figure 5a, all BrRAP2.4 proteins were able to assemble with the BPM protein from Arabidopsis, while GST alone was not.

Because AtRAP2.4^noPEST^ and BrRAP2.4-1^noPEST^ were more stable in the cell-free degradation assays, we were interested in testing whether the PEST motif is involved in assembling with a BPM protein. Pulldown assays showed that loss of the PEST motif decreases assembly of His:AtBPM3 with both RAP2.4^noPEST^ proteins (Figure 5b,c). Representative Western blots are shown in Supplementary Figure S6. We also tested the PEST motif alone, and could demonstrate that it is sufficient to interact with a BPM protein (Figure 5d). 

The change in interaction with AtBPM3 is in agreement with the increased stability of the two RAP2.4^noPEST^ proteins. In addition, these findings strongly support the notion that the PEST motif is required for normal assembly with a BPM protein, and that the increased stability of RAP2.4^noPEST^ proteins is likely a consequence of poor assembly with the CRL3 substrate adaptor. 

We had previously shown that AtRAP2.4 interacts with CUL3 [19], and tested this also for GST:BrRAP2.4-1 using total native protein extract from Arabidopsis wild type plants for pulldown assays. As shown in the Supplementary Figure S7, GST:BrRAP2.4-1 is able to precipitate CUL3 from the plant extract, providing strong evidence that the Brassica protein can assemble into a CRL3^BPM^ complex in planta.

### 3.7. BrRAP2.4 Proteins Are Located in the Nucleus

**Figure 6:**
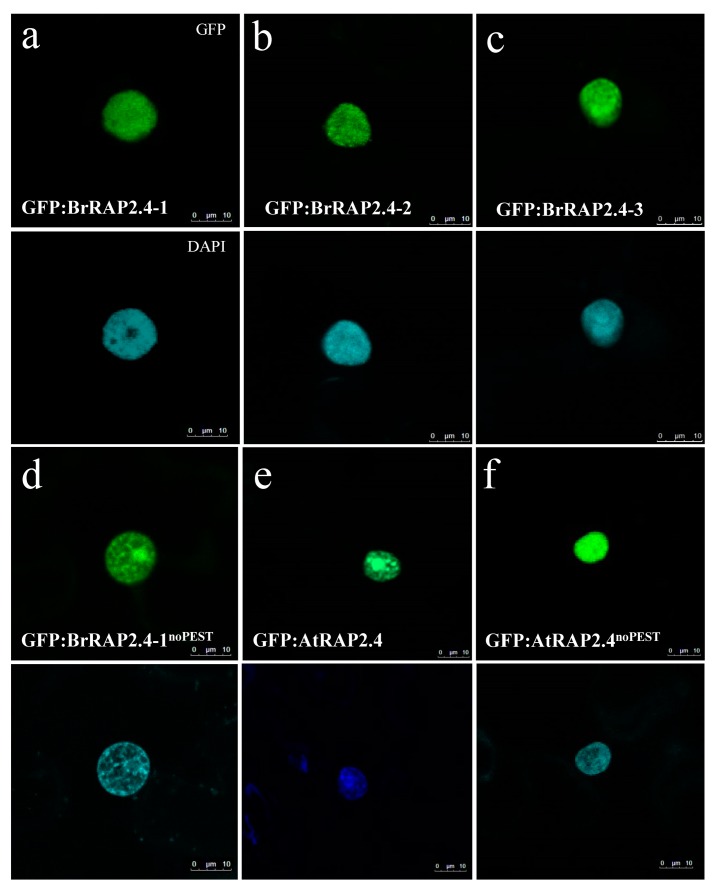
Nuclear Localization of GFP:RAP2.4 proteins in transient expression assays using tobacco leaves. (**a**–**c**) BrRAP2.4-1 to -3. (**d**) BrRAP2.4-1^noPEST^. (**e**) and (**f**) AtRAP2.4 and AtRAP2.4^noPEST^. Upper part always: confocal pictures showing GFP fluorescence, lower part always: confocal images showing DAPI fluorescence to indicate localization of the nucleus.

Transient expression analysis of GFP-tagged BrRAP2.4 proteins in *N. benthamiana* showed that the three BrRAP2.4 proteins are located in the nucleus (Figure 6a–c). In addition, deletion of the PEST motif in BrRAP2.4-1 and AtRAP2.4 does not affect their subcellular localization (Figure 6d,f), indicating that a main function of the motif is likely to facilitate assembly with BPM proteins to control RAP2.4 stability. 

### 3.8. BrRAP2.4-1 Overexpressing Plants Display Increased Salt Tolerance

**Figure 7:**
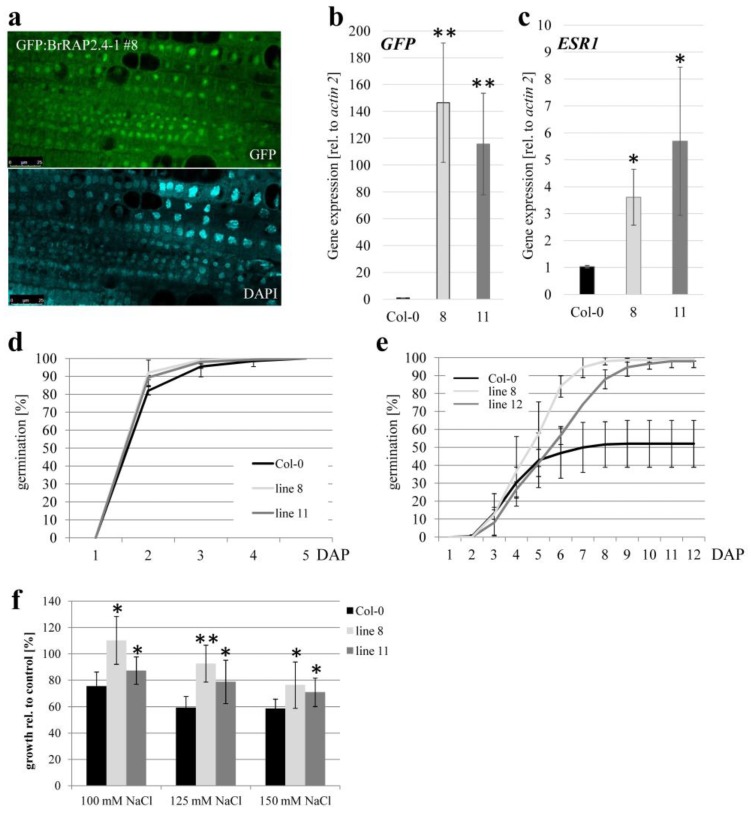
*BrRAP2.4-1* expression in Arabidopsis causes increased salt tolerance. (**a**) Confocal pictures of one of the transgenic Arabidopsis lines (#8) expressing GFP:BrRAP2.4-1 under the control of a 35S promoter. Pictures were taken on root tissue of 10-day old seedlings. Upper half: GFP image, lower half: DAPI staining. (**b**) qRT-PCR showing GFP-expression in two of the transgenic Arabidopsis lines (#8 and #11) but not in the wild type Col-0 control. *n* = 3 biological replicates, double asterisks indicate ANOVA test *p* < 0.01 significance. (**c**) The two Arabidopsis lines #8 and #11 showed significantly higher levels of *ESR1* expression. *n* = 3, asterisk indicate p<0.05 ANOVA test. (**d**) Germination of #8 and #11 seeds is not altered when compared to wild type Col-0 seeds on basic ATS medium. (**e**) Addition of 150 mM NaCl to the medium shows that seeds from lines #8 and #11 germinated earlier when compared to Col-0. Experiments in (**d**) and (**e**) were repeated 4 times, each time with at least 30 seeds per genetic background. Differences in (**e**) between Col-0 and the lines #8 and #11 were significant (*p* < 0.05) from day 7 on, based on an ANOVA test. (**f**) Lines #8 and #11 are less inhibited in primary root growth compared to wild type when grown on 150 mM NaCl. *n* = 3 biological replicates with at least 10 plants per trial. Asterisks indicate significance (* *p* < 0.05; ** *p* < 0.01; ANOVA test). DAP, days after plating.

Because BrRAP2.4-1 was regulated by different stress conditions, and AtRAP2.4 had been shown to alleviate abiotic stress in Arabidopsis [7,8], we decided to focus on this protein for in planta studies. Several independent *A. thaliana* transgenic lines were generated that stably overexpressed GFP:BrRAP2.4-1 under the control of a 35S promoter. Out of 45 independent transgenic plants identified, 25 showed some GFP expression when analyzed with a confocal microscope. In all cases, the GFP-fusion proteins were located to the nucleus (Figure 7a). However, the overall expression was always low, and we were not able to immunodetect GFP-fusion protein in these plants. This may be in part due to the observed instability of BrRAP2.4-1; but even treatment with MG132 did not lead to a detectable protein (Raed Al-Saharin, personal communication). 

To first confirm expression of the transgene, cDNAs were generated from five independent lines and the Col-0 wild type. A basic PCR on these cDNAs confirmed *GFP:BrRAP2.4-1* expression in the transgenic plants, while no product was generated in Col-0 (Supplementary Figure S8). Two of the transgenic lines, GFP:BrRAP2.4-1 #8 and #11, that had the strongest GFP signals under the confocal microscope, showed strong *GFP* expression in qRT-PCR experiments, while this was not the case for the wild type (Figure 7b). In general, the number #8 line had a slightly stronger *GFP* expression then #11. 

So far only one target gene has been described, *ENHANCER OF SHOOT REGENERATION 1 (ESR1)*, another ERF/AP2 transcription factor that is directly controlled by AtRAP2.4 [12]. As shown in Figure 7c, *ESR1* is slightly up-regulated in both GFP:BrRAP2.4-1 #8 and #11. The mild increase in the transgenic plants likely contributed to the comparably low GFP:BrRAP2.4-1 protein levels. However, the change was significant and provided strong evidence that the BrRAP2.4-1 proteins are transcriptionally active in Arabidopsis, and that they can also target *ESR1*. 

We further tested different stress conditions such as drought, heat, and salt, but could not observe any obvious changes for drought and heat stress sensitivities in the two BrRAP2.4-1 overexpressing Arabidopsis lines (Raed Al-Saharin, personal communication). However, germination assays in the presence of salt (150 mM NaCl) showed a significantly higher germination rate in the two transgenic lines when compared to the wild type (Figure 7d,e). In addition, root elongation assays in the presence of NaCl also showed that the transgenic plants performed better than the wild type in the presence of salt (Figure 7f). These findings showed that even mild increases in BrRAP2.4-1 can increase salt tolerance in Arabidopsis, at least at early developmental stages. 

## 4. Discussion

In this work we showed that *B. rapa* encodes for and expresses three genes highly related to Arabidopsis RAP2.4. All three of these genes are expressed with different patterns in *B. rapa*. *BrRAP2.4-1* expression is mostly stress inducible, but otherwise the gene has low basal levels of transcription compared to the other *BrRAP2.4s*. In contrast, levels of *BrRAP2.4-3* exhibit little to no stress regulation, despite having the strongest expression across the tissues tested, especially in siliques and flowers. Finally, *BrRAP2.4-2* has an intermediate strength of expression, and like *BrRAP2.4-3* poorly responds to stress exposure. Cloning the *BrRAP2.4* genes showed that two deviated from the publicly annotated *B. rapa* sequences, most pronounced for *BrRAP2.4-1*, which is identical to an annotated *B. napus* protein. Because *B. napus* contains genomes from the parent species *B. rapa* and *B. oleracea*, we consider it likely that the annotated *B. napus* gene reflects the *B. rapa* ancestry. It is also probable that the A genome in our *B. rapa* variety is closely related to the *B. napus* reference genome [45].

It is currently difficult to provide an exact function for the different BrRAP2.4 proteins. The specific expression patterns of *BrRAP2.4-1* in *B. rapa* suggested that the protein may have a role in stress alleviation and wound response, similar to what has been described for the Arabidopsis ortholog [5,6,7]. In fact, the mildly increased levels of the GFP:BrRAP2.4-1 fusion protein were already sufficient to improve salt tolerance at the germination and seedling level. It will be of interest to see whether its overexpression in *B. rapa* also results in increased tolerance towards salt and other abiotic stress factors, such as drought or heat as described earlier [7,8,9]. Because the overexpression of *AtRAP2.4* in tomato, tobacco, and rapeseed caused comparable wound and cell dedifferentiation responses, the basic function of this protein appears to be conserved among the different plant species [4,5]. Since *BrRAP2.4-1* expression is wound-inducible, it may also fulfill this role in *B. rapa.* AtRAP2.4 is part of the A6 ERF/AP2 subfamily of which several members have been brought into context with cell dedifferentiation [38]. It is therefore possible that BrRAP2.4-2 and -3 may also participate in this process, despite their lack of wound and stress-dependent expression in seedlings. It is also noteworthy that cell-dedifferentiation and drought tolerance were mainly connected with very high levels of *AtRAP2.4* expression under the control of a constitutive promoter [4,5,7]. In our plants, expression levels were comparably mild. These increased levels of protein may not be sufficient to induce the previously described phenotypes, especially since the BrRAP2.4-1 protein is instable and is likely quickly degraded in planta.

The high level of *BrRAP2.4-3* in flowers and siliques may indicate that this protein has a critical role in reproductive processes. For example, pollen and seeds undergo desiccation as part of their maturation process. This is a developmental program that prepares the corresponding cells and organs for a dramatic reduction in water content [46]. It could be of interest to down-regulate the expression of *BrRAP2.4-3* in flowers and seeds to see whether this impacts reproductive processes.

RAP2.4 proteins may also be involved in trehalose-6-phosphate metabolism, a carbohydrate that is considered as a central molecule involved in providing the cell with critical information on its metabolic status, and as a stress-related compound [47,48,49]. It was previously published that several members of the A6 family in Arabidopsis, to which *AtRAP2.4* belongs, are in close chromosomal proximity to a *trehalose-6-phosphate phosphatase* (*TPP*) gene [6]. Curiously this also holds true for *BrRAP2.4-2* and *-3,* which are near a predicted *TPP* gene (*Bra008344* (chromosome A02 at nucleotide position 13,895,221 to 13897525), and *Bra015631* (chromosome A07 at nucleotide position 24,941,042 to 24943612), respectively). Although it remains open whether this has any functional relevance, and whether this type of genetic arrangement is restricted to the two Brassicaceae species, it is of interest to note that overexpression of AtRAP2.4 in *B. napus* did not only cause up-regulation in proline and γ-amino butyric acid levels, but also affected trehalose metabolism [13]. 

The findings that the BrRAP2.4 proteins assemble with AtBPM3, and that they are instable in a proteasome-dependent manner, make it likely that all three proteins are substrates of a CRL3^BPM^ E3 ligase in *B. rapa*. The situation of the BPM proteins is more complex here as this Brassica species has almost twice as many BPM proteins compared to Arabidopsis [24]. To better understand their roles in *B. rapa*, it may be important to test individual *BrBPM* expression patterns in the different tissues, and to investigate whether some of the *B. rapa BPMs* are regulated by stress. In this context it is also noteworthy that all RAP2.4 proteins investigated in this work contain PEST motifs, and that, at least for AtRAP2.4 and BrRAP2.4-1, deletion of this motif resulted in increased stability and reduced assembly with AtBPM3. PEST motifs were also described for AtWRI1, another CRL3^BPM^ target, and here the motif also proved to be critical for its stability [50]. However, the PEST motif in AtWRI1 has so far not been shown to directly facilitate interaction with BPMs, and appears to be more critical for stability control in the RAP2.4 proteins compared to AtWRI1. 

Since RAP2.4 proteins that lack the PEST motif are still degraded and can still interact with AtBPM3, though clearly reduced, these findings point out that additional motifs are probably present in BrRAP2.4-1 that play a role in controlling its stability and assembly with BPMs. In fact, one other motif was previously described to facilitate substrate assembly with the human BPM ortholog SPOP [51]. SPOP substrates contain a short peptide called the SPOP-Binding Consensus (SBC) motif with five amino acids often enriched in either serine or threonine residues [51]. Recently, it was also confirmed that this motif is conserved among plants and animals [29]. A clear SBC motif is also present in AtRAP2.4 (PSSSS, amino acid residues 36-40), but we did not observe that its deletion dramatically impacted either stability or assembly with AtBPM3 (Supplementary Figure S9a–c). Even with the deletion of both the SBC motif and the PEST motif from AtRAP2.4, no drastic change was observed in stability when compared to the PEST deletion only (Supplementary Figure S9d). Interestingly, neither BrRAP2.4-1 nor the other two BrRAP2.4 proteins have an obvious SBC motif, so it will be interesting to understand what other sites, besides the PEST motif, contribute to the assembly with BPM proteins in these Brassica proteins. 

## 5. Conclusions

*B. rapa* is a commercially relevant crop. The specific subspecies we worked with (‘oleifera’) is very similar to *B. napus*, and can be used as a good model for Canola plants. This is further supported by our finding that *BrRAP2.4-1* cloned from R-o-18 is identical to a *B. napus* gene. Subspecies have been cultivated for a long time in different cultures, and are bred not only for seed oil, like canola, but also directly for root or leaf consumption. Good examples here are turnip (*B. rapa* subsp. *rapa*) for root, and napa cabbage (*B. rapa* subsp. *pekinensis*) or Japanese mustard spinach (*B. rapa* var. *perviridis*) for leaf. If altered levels of the RAP2.4s and BPMs in R-o-18 have beneficial impacts for the plants, one can likely transfer this knowledge directly to Canola and other *B. rapa* subspecies with similar results.

## Supplementary Materials

The following are available online at https://www.mdpi.com/2073-4409/8/4/336/s1, Figure S1: qRT-PCR expression analysis of BrRAP2.4-1, -2, and -3 in different tissues of *B. rapa* plants. Figures S2–S4: Sequence information on BrRAP2.4-1. Figure S5: DNA and amino acid sequences of BrRAP2.4-2 and BrRAP2.4-3. Figure S6: Representative blots for Figure 5b–c quantification. Figure S7: Pulldown analysis showing that GST:AtRAP2.4 and GST:BrRAP2.4-1 can pulldown AtCUL3 from plants extracts, while GST alone can not. Figure S8: Basic expression analysis of GFP:BrRAP2.4-1 in Arabidopsis. Figure S9: Deletion of the SBC motif in AtRAP2.4 does not significantly alter stability or interaction with AtBPM3. Table S1: List of Primers used in this work.
