# Supplementary material for: Characterization of Brassica rapa RAP2.4-Related Proteins in Stress Response and as CUL3-Dependent E3 Ligase Substrates
Source: Cells. 2019 Apr 10;8(4):336. doi: 10.3390/cells8040336 (PMC6523098; doi:10.3390/cells8040336)
Supplement: Supplementary file 1 [file cells-08-00336-s001.pdf]

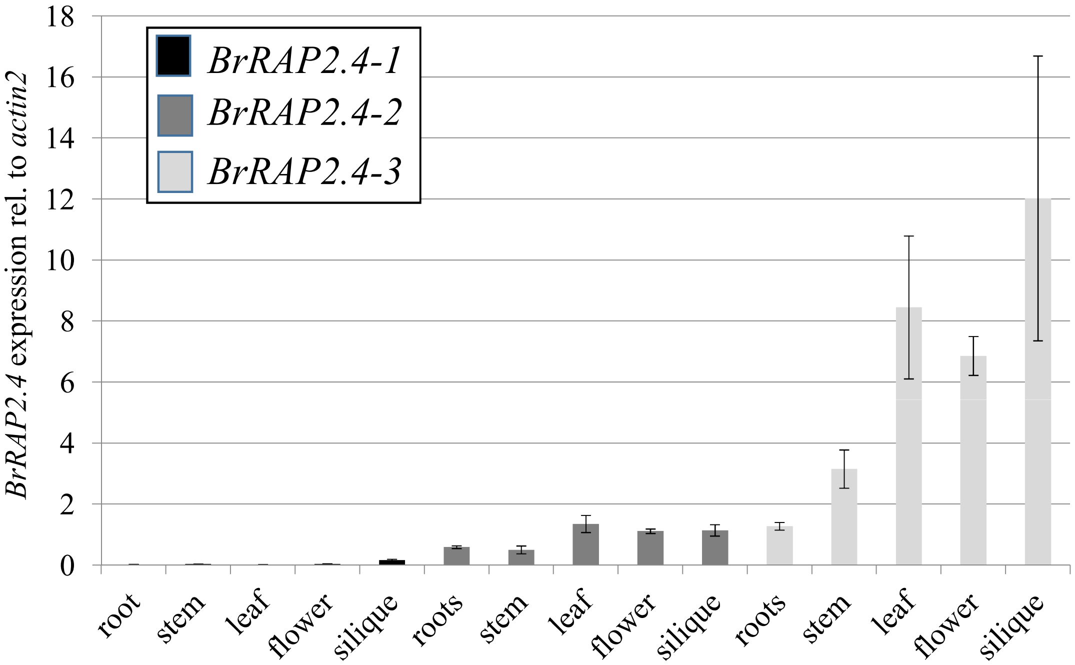


***Figure S1.*** qRT-PCR expression analysis of *BrRAP2.4-1*, *-2*, and *-3* in different tissues of *B. rapa* plants. Shown is a technical replicate. Standard bars represent standard error.

atggaagctgctatgaatttgaacagtagcagaacgtttcaacaaccagattcgtttggtggt

**M E A A M N L N S S R T F Q Q P D S F G G**

ggtggcgaactcatggaagcgcttctacctttatcaaaagcgtttccgactctccttccgcg

**G G E L M E A L L P F I K S V S D S P S A**

tccgcgtctgcgtttattaatcccgcagcgtctgcgtttcctcttcccaccttccgcgattac

**S A S A F I N P A A S A F P L P T F R D Y**

aacccggaacactatctgacccaaccgtttccgtacgggtcggatcttcagcaaaccgggtca

**N P E H Y L T Q P F P Y G S D L Q Q T G S**

ctaatcgggctcaacaacctctcctcttcccaaatccaccagatccaatctcagatccaccac

**L I G L N N L S S S Q I H Q I Q S Q I H H**

aaccatcctctccctcccaccagatccaacctcaacctcagccccaagccgttactgatgaag

**N H P L P P T R S N L N L S P K P L L M K**

caacctggagtcgccggatcctgcttcgcctacggagctccgccgaagccggcgaagctgtac

**Q P G V A G S C F A Y G A P P K P A K L Y**

agaggcgtgaggcagcgtcactggggaaaatgggtggcggagatccgtttgccgaggaaccgg

**R G V R Q R H W G K W V A E I R L P R N R**

actcgtctctggcttgggacgttcgacacggcggaggaagctgcgttggcctacgatacggcg

**T R L W L G T F D T A E E A A L A Y D T A**

gcgtttaagctgcgcggcgatttcgcccggcttaacttccctaacctgcgtcacgacggatct

**A F K L R G D F A R L N F P N L R H D G S**

cgcatcggaggcgagttcggcgagtataaacctcttcactccactgtcgacgccaagctcgaa

**R I G G E F G E Y K P L H S T V D A K L E**

gctatttgtaagagtatggcggagacggagaaacaggagaagacgacgaaggcgtcgaagaaa

**A I C K S M A E T E K Q E K T T K A S K K**

cgcgcatcgacggcggcagtcaaggcggaggagaattcgaattcgatcggtgagtctccaccg

**R A S T A A V K A E E N S N S I G E S P P**

atgacggagctcgttgagtccgccggatcttcgccgttgtcggagttgacgttcgccgacgcg

**M T E L V E S A G S S P L S E L T F A D A**

gaggagcagccgcagtggaacgagaccttcgcgttggagaagtatccgtcgtacgagatcgat

**E E Q P Q W N E T F A L E K Y P S Y E I D**

tgggattcgattttgccttga

**W D S I L P -**

***Figure S2.*** Alignment of DNA and amino acid sequence of BrRAP2.4-1

**a**

```
Annotated-Bra003659    ATGGAAGCTGCTATGAATTTGAACAGTAGCAGAACGTTTCAACAACCAGATTCGTTTGGTGGTGGTGGCGGTGAACTC
Cloned-Bra003659       ATGGAAGCTGCTATGAATTTGAACAGTAGCAGAACGTTTCAACAACCAGATTCGTTTGGTGGTGGTGGC---GAACTC
                       *********************************************************************   ******

Annotated-Bra003659    ATGGAAGCGCTTCTACCTTTTATCAAAAGCGTTTCCGACTCTCCTTCCGCGTCCGCGTCTGCGTTTATTAATCCCGCA
Cloned-Bra003659       ATGGAAGCGCTTCTACCTTTTATCAAAAGCGTTTCCGACTCTCCTTCCGCGTCCGCGTCTGCGTTTATTAATCCCGCA
                       ******************************************************************************

Annotated-Bra003659    GCGTCTGCGTTCCCTCTTCCCACCTTCCCCGGATACTACCCGGAA------CTGACCCAACTGTTTCCGTACGGGTCG
Cloned-Bra003659       GCGTCTGCGTTTCCTCTTCCCACCTTCCGCGATTACAACCCGGAACACTATCTGACCCAACCGTTTCCGTACGGGTCG
                       *********** *************** **   *** ********      ********** ****************

Annotated-Bra003659    GATCTTCAGCAAACCGGATCACTAATCGGGCTCAACAACCTCTCCCCTACCCAAATCCACCAGATCCAATCTCAGATC
Cloned-Bra003659       GATCTTCAGCAAACCGGGTCACTAATCGGGCTCAACAACCTCTCCTCTTCCCAAATCCACCAGATCCAATCTCAGATC
                       ***************** *************************** ** *****************************

Annotated-Bra003659    CACCACAACCATCCTCTCCCTCCCACCAGATC------CAACCTCAGCCCCAAGCCGTTACTGATGAAGCAACCTGGA
Cloned-Bra003659       CACCACAACCATCCTCTCCCTCCCACCAGATCCAACCTCAACCTCAGCCCCAAGCCGTTACTGATGAAGCAACCTGGA
                       ********************************      ****************************************

Annotated-Bra003659    GTCGCCGGATCCTGCTTCGCAGACGGTGCTCCGCCGAAGCCGGCGAAGCTATACAGAGGCGTGAGGCAGCGTCACTGG
Cloned-Bra003659       GTCGCCGGATCCTGCTTCGCCTACGGAGCTCCGCCGAAGCCGGCGAAGCTGTACAGAGGCGTGAGGCAGCGTCACTGG
                       ********************  **** *********************** ***************************

Annotated-Bra003659    GGAAAATGGGTGGCGGAGATCCGTTTGCCGAGGAACCGGACTCGTCTCTGGCTTGGGACGTTCGACACGGCGGAGGAA
Cloned-Bra003659       GGAAAATGGGTGGCGGAGATCCGTTTGCCGAGGAACCGGACTCGTCTCTGGCTTGGGACGTTCGACACGGCGGAGGAA
                       ******************************************************************************

Annotated-Bra003659    GCTGCGCTGGCCTATGATACGGCGGCGTTTAAGCTGCGCGGCGATTTCGCCCGGCTTAACTTCCCTAACCTGCGTCAC
Cloned-Bra003659       GCTGCGTTGGCCTACGATACGGCGGCGTTTAAGCTGCGCGGCGATTTCGCCCGGCTTAACTTCCCTAACCTGCGTCAC
                       ****** ******* ***************************************************************

Annotated-Bra003659    GACGGATCTCGCATCGGAGGCGAGTTCGGCGAGTATAAACCTCTTCACTCCACTGTCGACGCCAAGCTCGAAGCTATT
Cloned-Bra003659       GACGGATCTCGCATCGGAGGCGAGTTCGGCGAGTATAAACCTCTTCACTCCACTGTCGACGCCAAGCTCGAAGCTATT
                       ******************************************************************************

Annotated-Bra003659    TGTAAGAGTATGGCGGAGACGGAGAAACAGGAGAAGACGACGAAGGCGTCGAAGAAACGCGCATCGACGGCGGCAGTC
Cloned-Bra003659       TGTAAGAGTATGGCGGAGACGGAGAAACAGGAGAAGACGACGAAGGCGTCGAAGAAACGCGCATCGACGGCGGCAGTC
                       ******************************************************************************

Annotated-Bra003659    AAGGCGGAGGAGAATTCGAATTCGATCGGTGAGTCTCCACCGGTGACGGAGTTCGTTGAGTCCGCCGGATCTTCGCCG
Cloned-Bra003659       AAGGCGGAGGAGAATTCGAATTCGATCGGTGAGTCTCCACCGATGACGGAGCTCGTTGAGTCCGCCGGATCTTCGCCG
                       ****************************************** ******** **************************

Annotated-Bra003659    TTGTCGGAGTTGACGTTCGCCGACGCGGAGGAGCAGCCGCAGTGGAACGAGACCTTCGCGTTGGAGAAGTATCCGTCG
Cloned-Bra003659       TTGTCGGAGTTGACGTTCGCCGACGCGGAGGAGCAGCCGCAGTGGAACGAGACCTTCGCGTTGGAGAAGTATCCGTCG
                       ******************************************************************************

Annotated-Bra003659    TACGAGATCGATTGGGATTCGATTTTGCCTTGA
Cloned-Bra003659       TACGAGATCGATTGGGATTCGATTTTGCCTTGA
                       *********************************
```

**Deletion**
**Insertion**
**Synonymous different**
**Non-synonymous different**

**b**

```
Annotated-Bra003659    MEAAMNLNSSRTFQQPDSFGGGGGELMEALLPFIKSVSDSPSASASAFINPAASAFPLPTFPGYYPE--LTQLFPYGS
Cloned-Bra003659       MEAAMNLNSSRTFQQPDSF-GGGGELMEALLPFIKSVSDSPSASASAFINPAASAFPLPTFRDYNPEHYLTQPFPYGS
                       ******************* ***************************************** .* **  *** *****

Annotated-Bra003659    DLQQTGSLIGLNNLSPTQIHQIQSQIHHNHPLPPTRS--NLSPKPLLMKQPGVAGSCFADGAPPKPAKLYRGVRQRHW
Cloned-Bra003659       DLQQTGSLIGLNNLSSSQIHQIQSQIHHNHPLPPTRSNLNLSPKPLLMKQPGVAGSCFAYGAPPKPAKLYRGVRQRHW
                       ***************.:********************  ******************** ******************

Annotated-Bra003659    GKWVAEIRLPRNRTRLWLGTFDTAEEAALAYDTAAFKLRGDFARLNFPNLRHDGSRIGGEFGEYKPLHSTVDAKLEAI
Cloned-Bra003659       GKWVAEIRLPRNRTRLWLGTFDTAEEAALAYDTAAFKLRGDFARLNFPNLRHDGSRIGGEFGEYKPLHSTVDAKLEAI
                       ******************************************************************************

Annotated-Bra003659    CKSMAETEKQEKTTKASKKRASTAAVKAEENSNSIGESPPVTEFVESAGSSPLSELTFADAEEQPQWNETFALEKYPS
Cloned-Bra003659       CKSMAETEKQEKTTKASKKRASTAAVKAEENSNSIGESPPMTELVESAGSSPLSELTFADAEEQPQWNETFALEKYPS
                       ****************************************:**:**********************************

Annotated-Bra003659    YEIDWDSILP
Cloned-Bra003659       YEIDWDSILP
                       **********
```

**Deletion**
**Insertion**
**Different**

***Figure S3.*** Sequence information on BrRAP2.4-1 **a** DNA sequence alignment of annotated and cloned BrRAP2.4-1. **b** amino acid sequence alignment of annotated and cloned BrRAP2.4-1

```
AtRAP2.4                  MAAAMNLYTCSRSFQ------DSGGELMDALVPFIKSVSDSPSSSSAASASAFLHP--SA
BnaA07g20720D             MEAAMNL-NSSRTFQQPDSF-GGGGELMEALLPFIKSVSDSPS----ASASAFINPAASA
Cloned-Bra003659          MEAAMNL-NSSRTFQQPDSF-GGGGELMEALLPFIKSVSDSPS----ASASAFINPAASA
Annotated-Bra003659       MEAAMNL-NSSRTFQQPDSFGGGGELMEALLPFIKSVSDSPS----ASASAFINPAASA
                          * ***** ..**:**      ..*****:**:***********    ******::*  **
AtRAP2.4                  FSLPPLPGYYPDSTFLTQPFSYGSDLQQTGSLIGLNNLSSSQIHQIQSQIH--HPLPPTH
BnaA07g20720D             FPLPTFRDYNPEH-YLTQPFPYGSDLQQTGSLIGLNNLSSSQIHQIQSQIHHNHPLPPTR
Cloned-Bra003659          FPLPTFRDYNPEH-YLTQPFPYGSDLQQTGSLIGLNNLSSSQIHQIQSQIHHNHPLPPTR
Annotated-Bra003659       FPLPTFPGYYPE---LTQLFPYGSDLQQTGSLIGLNNLSPTQIHQIQSQIHHNHPLPPTR
                          *.**.: .* *:   *** *.******************.:**********  ******.
AtRAP2.4                  HNNNNSFSNLLSPKPLLMKQSGVAGSCFAYGSGVPSKPTKLYRGVRQRHWGKWVAEIRLP
BnaA07g20720D             SNLN------LSPKPLLMKQPGVAGSCFAYGA--PPKPAKLYRGVRQRHWGKWVAEIRLP
Cloned-Bra003659          SNLN------LSPKPLLMKQPGVAGSCFAYGA--PPKPAKLYRGVRQRHWGKWVAEIRLP
Annotated-Bra003659       SN--------LSPKPLLMKQPGVAGSCFADGA--PPKPAKLYRGVRQRHWGKWVAEIRLP
                           *        **********.******** *:  *.**:*********************
AtRAP2.4                  RNRTRLWLGTFDTAEEAALAYDKAAYKLRGDFARLNFPNLRHNGSHIGGDFGEYKPLHSS
BnaA07g20720D             RNRTRLWLGTFDTAEEAALAYDTAAFKLRGDFARLNFPNLRHDGSRIGGEFGEYKPLHST
Cloned-Bra003659          RNRTRLWLGTFDTAEEAALAYDTAAFKLRGDFARLNFPNLRHDGSRIGGEFGEYKPLHST
Annotated-Bra003659       RNRTRLWLGTFDTAEEAALAYDTAAFKLRGDFARLNFPNLRHDGSRIGGEFGEYKPLHST
                          **********************.**:****************:**.***:*********:
AtRAP2.4                  VDAKLEAICKSMAETQKQDKSTKSSKKREKKVSSPDLSEKVKAEENSVSIGGSPPVTEFE
BnaA07g20720D             VDAKLEAICKSMAETEKQEKTTKASKKRASTAA-------VKAEENSNSIGESPPMTELV
Cloned-Bra003659          VDAKLEAICKSMAETEKQEKTTKASKKRASTAA-------VKAEENSNSIGESPPMTELV
Annotated-Bra003659       VDAKLEAICKSMAETEKQEKTTKASKKRASTAA-------VKAEENSNSIGESPPVTEFV
                          ***************:**:*:**:**** ...:       ******* *** ***:**:
AtRAP2.4                  ESTAGSSPLSDLTFADPEEPPQWNETFSLEKYPSYEIDWDSILA
BnaA07g20720D             ES-AGSSPLSELTFADAEEQPQWNETFALEKYPSYEIDWDSILP
Cloned-Bra003659          ES-AGSSPLSELTFADAEEQPQWNETFALEKYPSYEIDWDSILP
Annotated-Bra003659       ES-AGSSPLSELTFADAEEQPQWNETFALEKYPSYEIDWDSILP
                          ** *******:*****.** *******:***************.
```

***Figure S4.*** Amino acid sequence alignment of AtRAP2.4, BnaA07g20720D, and the two annotated and cloned sequences for BrRAP2.4-1

**a**

```
atggaagctgctatgaatatgtacaatagcagaacggttcaccaatcagattcgtttggtggt
 M  E  A  A  M  N  M  Y  N  S  R  T  V  H  Q  S  D  S  F  G  G
ggtgaactcatggaagcgcttgtaccttttatcaaaagcgtttccacctcttcttctcctcct
 G  E  L  M  E  A  L  V  P  F  I  K  S  V  S  T  S  S  S  P  P
cctcccgcgtccgcgtctgcgtttattaaccccgcagcgtctgcgttctctcttcctaccttc
 P  P  A  S  A  S  A  F  I  N  P  A  A  S  A  F  S  L  P  T  F
cccggatatcacccggaacacttcatgacccaaccgtttacatacgggtcggatcttaaccaa
 P  G  Y  H  P  E  H  F  M  T  Q  P  F  T  Y  G  S  D  L  N  Q
accgggtcatcattaatcgggctaaacaacctctcttcttcccaaatccaccagatccaatct
 T  G  S  S  L  I  G  L  N  N  L  S  S  S  Q  I  H  Q  I  Q  S
cagatccaccacaaccatcctctcctcaggccaaagcctttactcatgaagcaacccggagtc
 Q  I  H  H  N  H  P  L  L  R  P  K  P  L  L  M  K  Q  P  G  V
gccggatcttgtttcccggcgaagccgacgaagctgtacagaggcgtgaggcaacgtcactgg
 A  G  S  C  F  P  A  K  P  T  K  L  Y  R  G  V  R  Q  R  H  W
ggaaaatgggtggcggagatccgtttgccgaggaaccgtactcgtctctggctaggaacgttc
 G  K  W  V  A  E  I  R  L  P  R  N  R  T  R  L  W  L  G  T  F
gacacggcggaggaagcagccttggcttacgacaaggcggcgtataagctgcgcggcgatcac
 D  T  A  E  E  A  A  L  A  Y  D  K  A  A  Y  K  L  R  G  D  H
gcccggcttaacttccctaacttacgtcacaacggttcccacatcggcggcgagttcggcgag
 A  R  L  N  F  P  N  L  R  H  N  G  S  H  I  G  G  E  F  G  E
tacaagcctcttcactcaacggtcgacgctaagctcgaagctatttgtcagagcatggcggag
 Y  K  P  L  H  S  T  V  D  A  K  L  E  A  I  C  Q  S  M  A  E
acgcagaaacaggacaaaacagcgaaagcttcgaagaaacgtgcctcgacggtgaagaaaact
 T  Q  K  Q  D  K  T  A  K  A  S  K  K  R  A  S  T  V  K  K  T
gagaaggttgatttgtcggagaaagtcagtccggtgacggagttcgttgagtccgccgggtct
 E  K  V  D  L  S  E  K  V  S  P  V  T  E  F  V  E  S  A  G  S
tcgccgttgtcggagctgacgttcgctgacaccgaggagcagccgcggtggaacgagaccttc
 S  P  L  S  E  L  T  F  A  D  T  E  E  Q  P  R  W  N  E  T  F
tcgttggagaagtatccgtcttacgagatcgactgggattcgatactgtcttga
 S  L  E  K  Y  P  S  Y  E  I  D  W  D  S  I  L  S  -
```

**b**

```
atggaagctgctttgaatatgtacaatagcataacgtttcaacagccagattcgtttggtggt
 M  E  A  A  L  N  M  Y  N  S  I  T  F  Q  Q  P  D  S  F  G  G
ggtgaactcatggaagcgcttgtcccttatataaatagcgtttccaattcttctccttatctc
 G  E  L  M  E  A  L  V  P  Y  I  N  S  V  S  N  S  S  P  Y  L
gcgtctgcgtttattcaacccgcagcgtctgcgtttcctccttctctacctaccttccccgct
 A  S  A  F  I  Q  P  A  A  S  A  F  P  P  S  L  P  T  F  P  A
tactacccggaagactattcaacgttcatgacccaaccgtttacttacgggtcggatcttcac
 Y  Y  P  E  D  Y  S  T  F  M  T  Q  P  F  T  Y  G  S  D  L  H
caaaccgggtcattaaccgggctcaaccacctctcttccagccaaactcatcctcttcctccc
 Q  T  G  S  L  T  G  L  N  H  L  S  S  S  Q  T  H  P  L  P  P
atgcaccatcagagccacaacaacaccttctcgaaccttctcagccctaagccgttactgatg
 M  H  H  Q  S  H  N  N  T  F  S  N  L  L  S  P  K  P  L  L  M
aagcagaccggtgccaccggatcttgtttcgcctacggtgctccggccaagccgacgaagctg
 K  Q  T  G  A  T  G  S  C  F  A  Y  G  A  P  A  K  P  T  K  L
tacagaggcgtgaggcagcgtcactggggaaatgggtggcggagatccgtttgccgaggaac
 Y  R  G  V  R  Q  R  H  W  G  K  W  V  A  E  I  R  L  P  R  N
cgtacccgtctctggcttgggacgttcgacacggcggaggaagctgcgttggcctacgacaag
 R  T  R  L  W  L  G  T  F  D  T  A  E  E  A  A  L  A  Y  D  K
gcggcgtataagctgcgtggcgatttcgcccggcttaacttccctaacctgcgtcacaatggg
 A  A  Y  K  L  R  G  D  F  A  R  L  N  F  P  N  L  R  H  N  G
tcccacatcggcgagttcggcgagtacaaaccgcttcactccaccgtcgacgccaagctcgaa
 S  H  I  G  E  F  G  E  Y  K  P  L  H  S  T  V  D  A  K  L  E
gctatatgccagagcatggcggaggcggagaaaaacggcaaaacgatgacaaaagcatcgaag
 A  I  C  Q  S  M  A  E  A  E  K  N  G  K  T  M  T  K  A  S  K
aaacgtgcctcgaagacggtttcatcgccggagaaagtcaaggcggagaataactcgaactcg
 K  R  A  S  K  T  V  S  S  P  E  K  V  K  A  E  N  N  S  N  S
gtcggtggatctccgccggtgacggagttcgttgagtccgccggttcttcgccgttgtcggac
 V  G  G  S  P  P  V  T  E  F  V  E  S  A  G  S  S  P  L  S  D
ttaacgttcgccgacactgaggagccgccgcagtggaacgagacgttctcgttggagaagtat
 L  T  F  A  D  T  E  E  P  P  Q  W  N  E  T  F  S  L  E  K  Y
ccgtcgtacgagatagattgggattcgatcctgtcttga
 P  S  Y  E  I  D  W  D  S  I  L  S  -
```

***Figure S5.*** DNA and amino acid sequence of **(a)** BrRAP2.4-2 and **(b)** BrRAP2.4-3

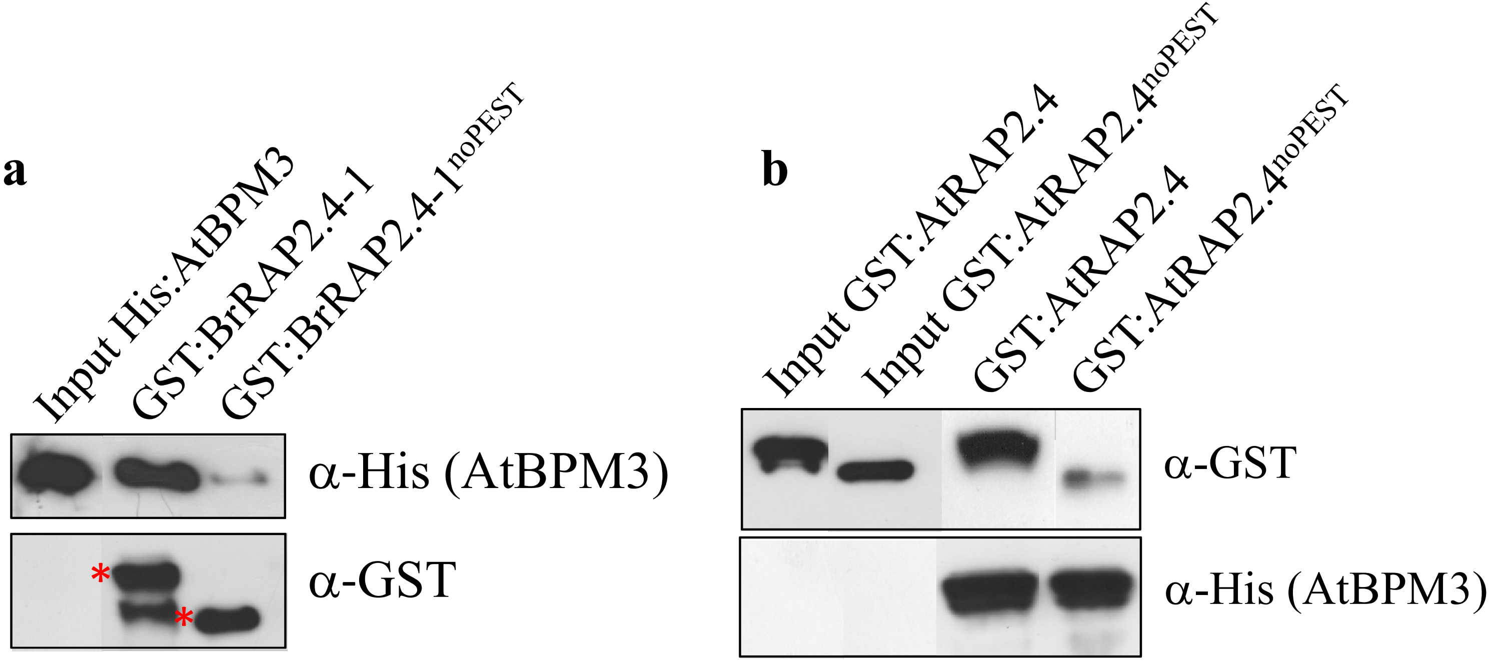


***Figure S6.*** Representative Western blots for Figure 5b-c quantification. **a**) Pulldown analysis with GST:BrRAP2.4 proteins on beads resulted in co-precipitation of eluted His:AtBPM3 protein, shows that the BrRAP2.4-1$^{noPEST}$ does not bind AtBPM3 as efficiently as full-length BrRAP2.4-1. **b**) Pulldown analysis with His:AtBPM3 protein on beads resulted in co-precipitation of eluted GST:AtRAP2.4 proteins shows that the AtRAP2.4$^{noPEST}$ does not bind AtBPM3 as efficiently as full-length AtRAP2.4.

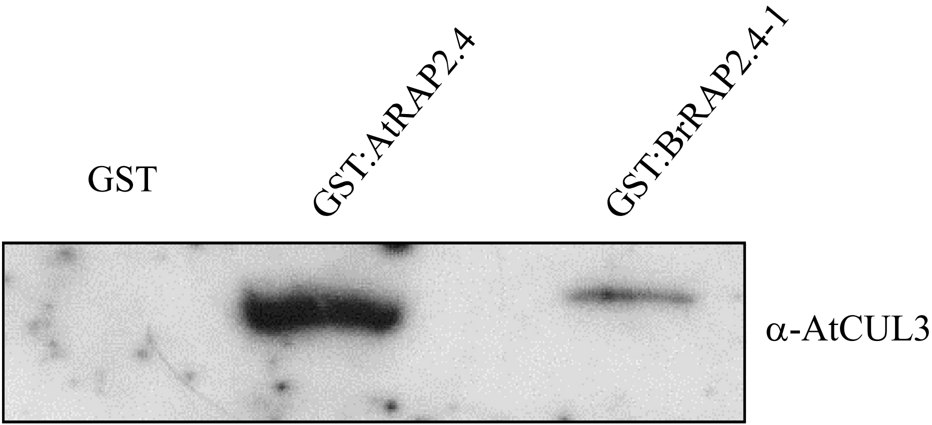


***Figure S7.*** Pulldown analysis showing that GST:AtRAP2.4 and GST:BrRAP2.4-1 can pulldown AtCUL3 from plants extracts, while GST alone can not.

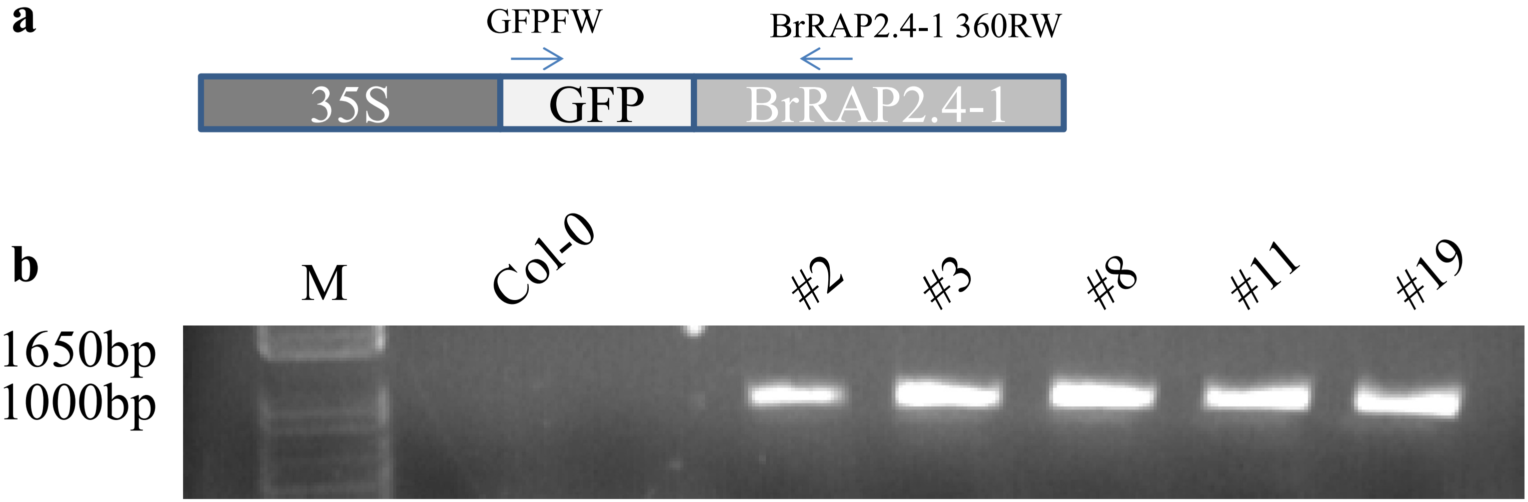


***Figure S8.*** Basic expression analysis of GFP:BrRAP2.4-1 in Arabidopsis. **a**) schematic drawing of the introduced construct. Arrows indicate primers used in (b). **b**) Total RNA was isolated from 10-day old seedlings, converted to cDNA, and used for a standard PCR. Product was detected in five transgenic lines (indicated by numbers), but not in Col-0 wild type. M, 100-bp DNA marker.

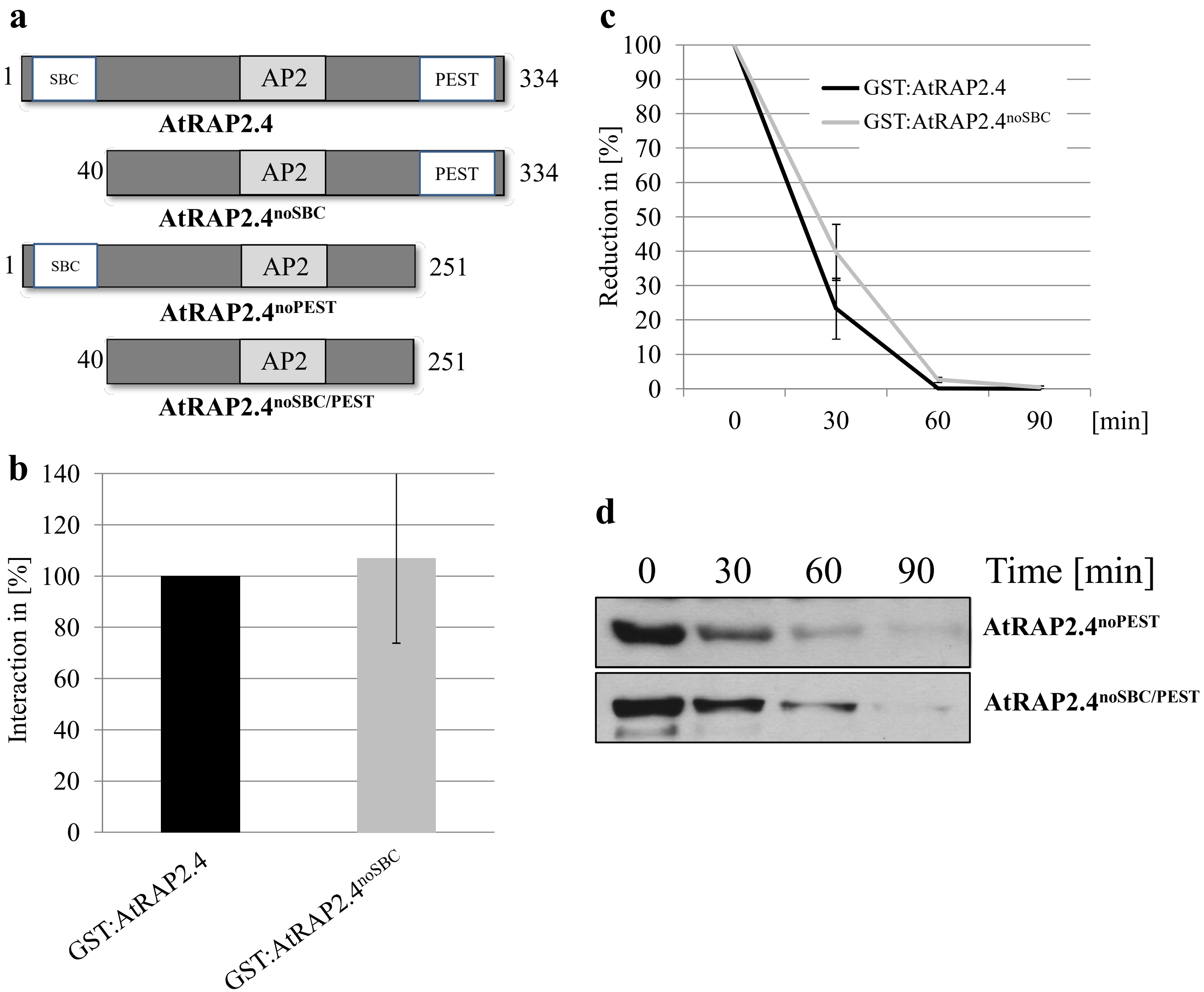


***Figure S9.*** Deletion of the SBC motif in AtRAP2.4 does not significantly alter stability or interaction with AtBPM3. **a)** Schematic drawing of AtRAP2.4 and AtRAP2.4$^{noSBC}$. **b**) Quantification of GST:AtRAP2.4 and GST:AtRAP2.4$^{noSBC}$ did not show any significant differences. **c**) AtRAP2.4$^{noSBC}$ degradation is only mildly but not significantly delayed in a cell free system. **d**) Cell-free degradation assays comparing stability of AtRAP2.4$^{noPEST}$ with AtRAP2.4$^{noSBC/PEST}$. Deletion of both, the SBC and the PEST motif, does not dramatically increase stability of the mutated AtRAP2.4$^{noSBC/PEST}$ protein when compared to AtRAP2.4$^{noPEST}$.

| **Table S1: List of Primers used in this work** | |
|---|---|
| **Primers used for BrRAP2.4 cloning** | |
| Bra003659FW | ATGGAAGCTGCTATGAATTTG |
| Bra003659RW | TCAAGGCAAAATCGAATCCC |
| Bra003659noPEST-RW | CTACTCCTGTTTCTCCGTCTC |
| Bra003659PEST-FW | CAGGAGAAGACGACGAAGGCG |
| Bra008343FW | ATGGAAGCTGCTATGAATATG |
| Bra008343RW | TCAAGACAGTATCGAATCCC |
| Bra015634FW | ATGGAAGCTGCTTTGAATATG |
| Bra015634RW | TCAAGACAGGATCGAATCCC |
| **Primer used for qRT-PCR Analysis** | |
| qRTAktinBra034778FW1 | GCTATGTATGTCGCTATC |
| qRTAktinBra034778RW1 | CATCTCCAGAGTCCAATA |
| Bra003659RT-FW | ACCTCTTCACTCCACTGT |
| Bra003659RT-RW | TCGTCGTCTTCTCCTGTT |
| Bra008343RT-FW | AACGGTTCACCAATCAGA |
| Bra008343RT-RW | GGAGAAGAAGAGGTGGAA |
| Bra015634RT-FW | GTTTCAACAGCCAGATTC |
| Bra015634RT-RW | GCGAGATAAGGAGAAGAA |
| **Primers used for checking Arabidopsis GFP:BrRAP2.4 transgenic lines** | |
| GFP-FW | ATGAGTAAAGGAGAAGAACTTTTC |
| Bra003659-360RW | GGGGCTGAGGTTGAGGTTGG |
| **Primers used for AtRAP2.4 cloning** | |
| AtRAP2.4 FW | ATGGCAGCTGCTATGAATTTG |
| AtRAP2.4 RW | CTAAGCTAGAATCGAATCCC |
| AtRAP2.4 noSBC-FW | ATGTCTGCAGCGTCTGCGTCT |
| AtRAP2.4 noPEST-RW | CTACGATTTGTCCTGTTTCTG |
